# A Comparison of Postoperative Early Enteral Nutrition with Delayed Enteral Nutrition in Patients with Esophageal Cancer

**DOI:** 10.3390/nu7064308

**Published:** 2015-06-02

**Authors:** Gongchao Wang, Hongbo Chen, Jun Liu, Yongchen Ma, Haiyong Jia

**Affiliations:** 1 School of Nursing, Shandong University, 44 Wenhua West Road, Jinan 250012, China; E-Mails: dabo1988@126.com (H.C.); liujunangel@163.com (J.L.); 2 Thoracic Surgery of Shandong Provincial Hospital, Shandong University, 9677 Jingshi Road, Jinan 250014, China; 3 School of Medicine, Shandong University, 44 Wenhua West Road, Jinan 250012, China; E-Mail: myc88587479@hotmail.com; 4 Department of Medicinal Chemistry, Key Laboratory of Chemical Biology (Ministry of Education), School of Pharmaceutica Sciences, Shandong University, 44 Wenhua West Road, Jinan 250012, China; E-Mail: 502378774@163.com

**Keywords:** early enteral nutrition, delayed enteral nutrition, esophageal cancer, postoperative complication

## Abstract

We examined esophageal cancer patients who received enteral nutrition (EN) to evaluate the validity of early EN compared to delayed EN, and to determine the appropriate time to start EN. A total of 208 esophagectomy patients who received EN postoperatively were divided into three groups (Group 1, 2 and 3) based on whether they received EN within 48 h, 48 h–72 h or more than 72 h, respectively. The postoperative complications, length of hospital stay (LOH), days for first fecal passage, cost of hospitalization, and the difference in serum albumin values between pre-operation and post-operation were all recorded. The statistical analyses were performed using the *t*-test, the Mann-Whitney U test and the chi square test. Statistical significance was defined as *p* < 0.05. Group 1 had the lowest thoracic drainage volume, the earliest first fecal passage, and the lowest LOH and hospitalization expenses of the three groups. The incidence of pneumonia was by far the highest in Group 3 (*p* = 0.019). Finally, all the postoperative outcomes of nutritional conditions were the worst by a significant margin in Group 3. It is therefore safe and valid to start early enteral nutrition within 48 h for postoperative esophageal cancer patients.

## 1. Introduction

Esophageal cancer is one of the common malignant tumors in the upper digestive system, which is currently listed as the world’s ninth most serious malignant disease [1,2,3]. Although curative surgery, radiotherapy and chemotherapy improve survival, the five-year survival rate of patients with esophageal cancer is still less than 50% [4]. Factors, such as preoperative diet restrictions, difficulty swallowing and the highly invasive nature of the treatment, cause patients to be prone to malnutrition after esophagectomy [5,6]. During the period that patients cannot take enough food orally, nutrient support plays an important role [7,8,9,10]. Nutrition support can be divided into parenteral nutrition and enteral nutrition. As complications of parenteral nutrition are more serious than those of enteral nutrition [11], thoracic surgery doctors use enteral nutrition as the main method of nutrition support. Past studies show that compared with those administered total parental nutrition, early postoperative total enteral nutrition (TEN) in high-risk surgical patients could reduce septic morbidity rates (TPN) [12,13,14,15]. Another study indicated that initiating enteral nutrition (EN) within 24 h had no advantage for the postoperative course of the patients with esophageal cancer compared with starting EN at 24–72 h [16]. For early enteral nutrition, opinions vary widely on the extent to which enteral nutrition should begin early. The term “early” was defined as EN started within three days after surgery [17]; however, more recently, “early” has been defined as EN started within 48 or 24 h after surgery [18]. The validity and safety of early EN after esophagectomy has remained controversial [7,16,19]. The aim of the present study is to verify the effectiveness and safety of early EN for the postoperative course and to determine an appropriate time to start enteral nutrition. 

## 2. Patients and Methods

### 2.1. Patient Selection 

Study subjects were selected from patients with esophageal cancer who were admitted for thoracic surgery in the Shandong provincial hospital, affiliated with Shandong University, between January 2013 and October 2014. The study protocol was approved by the Shandong university school of nursing science research project ethics committee (approval number 2012796). Esophageal cancer was diagnosed by gastroscopy. Patients suffering from diabetes or underlying cardiopulmonary diseases, whose body weight loss was more than 10% of the original, were excluded. All patients included in the study did not receive preoperative chemoradiotherapy. Moreover, the patients whose tumor staging was Stage IV (the cancer had spread to other parts of the body) were also excluded because they had lost the chance for curative surgery [20]. All patients received thoracotomy and extensive lymph node dissection during surgery. More specifically, the dissected lymph node included groups 2, 4, 7, 8, 9, 10 on the right side and groups 5, 6, 7, 8, 9, 10 on the left side. Mediastinal lymph node grouping was established according to the International Association for the Study of Lung Cancer (IASLC2009). A total of 208 patients were enrolled in the study. A total of 101 patients in the eastward department of thoracic surgery who received postoperative enteral nutrition within 48 h were included in Group 1. A total of 87 patients in the westward department who received postoperative enteral nutrition within 48–72 h were included in Group 2. For the patients of the two wards, who received early EN and who received very early EN was randomly determined. Twenty patients were included in Group 3 who suffered diarrhea, vomiting or had other adverse reactions after receiving early postoperative enteral nutrition who received enteral nutrition after 72 h. 

### 2.2. Methods 

The postoperative patients were given peripheral intravenous nutrition support before EN. Then the Enteral Nutritional Emulsion (TPF-T) (500 mL bag^−1^) made by Sino-Swed Pharmaceutical Corp. Ltd was used as the nutrition solution for enteral nutrition. The TPF-T was given by nutrition pump uniformly at the same temperature and speed through a nasojejunal tube, which was placed in the jejunum during the operation. The Fat Emulsion, Amino Acids (17) and Glucose (1%) Injection (1440 mL bag^−1^) made by Sino-Swed Pharmaceutical Corp. Ltd was used as the parenteral nutrition injected by central venous indwelling catheter. 

The day before received enteral nutrition, patients were given 150–200 mL 5% glucose and sodium chloride pumping at a speed of 20–25 mL h^−1^. At the same time, researchers observed the reaction of the patients. If there were no adverse reactions, such as abdominal distention, nausea, or vomiting, the nutrient solution would be started the next day. The total daily calories for all the patients should reach 125.52 kJ (30 kcal) kg^−1^ [21], and insufficient energy would be supplied by parenteral nutrition. On the first day of EN, the patients would be given a total of 500 mL nutrient solution, at a speed of 20–25 mL h^−1^. The EN dose was increased 500 mL every 24 h if there were no problems related to EN, and reached a maximum dose of 1500 mL every day. The patients (Group 3) who could not tolerate early enteral nutrition were given postoperative enteral nutrition after 72 h. Peripheral intravenous infusions of 5% glucose with electrolyte solutions were also performed to supply water and electrolytes in the three groups. EN continued until the anastomotic healing was confirmed by upper gastrointestinal barium, which would usually be done on the seventh postoperative day [22]. If one developed anastomotic leakage, the amount of time the patient received EN would be extended. 

### 2.3. Clinical Assessment

Clinical factors included age, sex, tumor stage according to the tumor-node-metastasis classification of the International Union against Cancer (7th edition) [20], pathological type, tumor location, surgical stress expressed by operation time (min) and blood loss during surgery (mL). Length of hospital stay (LOH), bowel movement recovery expressed as days for first fecal passage, cost of hospitalization, thoracic drainage volume(mL) were also recorded. The difference of albumin (g L^−1^) (Δalbumin), total protein (g L^−1^) (Δtotal protein) and absolute value of lymphocyte (×10^9^ L^−1^) (Δlymphocyte) between postoperative day 3 and pre-operation, and the difference in weight (Δ’ weight), albumin (g L^−1^) (Δ’ albumin), total protein (g L^−1^) (Δ’ total protein) and absolute value of lymphocytes (×10^9^ L^−1^) (Δ’ lymphocyte) between postoperative day 7 and pre-operation were important outcome indicators. Postoperative complications including infectious and noninfectious complications and mortality were also recorded. The infectious complications were those accompanying infections, such as pneumonia or wound infection, and the non-infectious complications were those such as anastomotic fistula or arrhythmia. 

### 2.4. Statistical Analysis

The statistical analyses were performed using the Mann-Whitney U test and adjusted chi-square test. The exact chi-square test was also used if individual cell size was less than 5 counts. The statistical significance was defined as *p* < 0.05. The quantitative data of normality underwent variance analysis and the quantitative data that did not meet normality criteria were analyzed using the rank sum test. Qualitative data were analyzed using the chi-square test. When multiple comparisons were needed, the rank sum test and the chi-square test inspection levels would correct to 0.0125 (0.05/4 = 0.0125) [23]. 

## 3. Results

### 3.1. Pre- and Peri-Operative Clinical Features

Among the 208 patients, 101 patients were categorized into Group 1, 87 patients were categorized into Group 2 and 20 patients were categorized into Group 3. There was no significant difference in mean age, sex, tumor stage, pathological type, tumor location, surgical stress expressed by operation time and blood loss, preoperative nutritional conditions expressed by body weight, or serum total protein values among the three groups. However, serum albumin values were significantly lower in Group 2 of the three groups (*p* = 0.001). Preoperative immune conditions expressed by the absolute value of lymphocyte were also comparable among the groups (Table 1). 

**Table 1:** Pre- and peri-operative clinical features among the three groups.

	Group1 (*n* = 101)	Group2 (*n* = 87)	Group3 (*n* = 20)	*p* value (One-way ANOVA)	
Age (years) ^a^	58.6 ± 7.5	59.5 ± 8.4	60.3 ± 9.7	0.602	
Body weight (kg) ^a^	65.2 ± 9.9	64.5 ± 10.4	64.7 ± 6.3	0.89	
Operative time (min) ^a^	212.7 ± 27.2	204.8 ± 20.0	213.3 ± 34.7	0.083	
Blood loss (mL )^a^	213.6 ± 21.9	206.7 ± 17.9	214.5 ± 26.7	0.055	
Albumin (g L^−1^) ^a^	42.3 ± 4.7	40.6 ± 3.7	44.2 ± 3.4	0.001	
Total protein (g L^−1^) ^a^	69.6 ± 5.4	68.0 ± 5.0	69.9 ± 4.9	0.086	
Absolute value of lymphocyte (×10^9^ L^−1^) ^a^	1.89 ± 0.51	1.85 ± 0.48	1.86 ± 0.32	0.808	
				***p* value (Chi square test)**	
Sex				0.509	
Male	90	77	16		
Female	11	10	4		
Stage				0.998	
0~ⅡB	60	52	12		
Ⅲ	41	35	8		
Pathological type				0.125	
Adenocarcinoma	4	0	1		
squamous carcinoma	97	87	19		
	**Group1 (*n* = 101)**	**Group2 (*n* = 87)**	**Group3 (*n* = 20)**	***p* value (Chi square test)**
Tumor location				0.173
upper thoracic portion	30	23	2	
mid-thoracic portion	36	42	10	
lower thoracic portion	35	22	8	

^a^ Data and mean ± standard deviation.

### 3.2. Postoperative Complications and Mortality

Postoperative complications were observed in 116 patients (55.77%) and there was a significant difference in whole complications among the three groups (*p* = 0.01). Using a partition of the chi-square method to do multiple comparisons, the result showed that there was a significant difference between Group 1 and Group 3 (*p* = 0.006 < 0.0125). Furthermore, postoperative complications were categorized into two groups: infectious and non-infectious. There was significant difference in the frequency of infectious complications; they were observed most frequently in Group 3 (*p* = 0.008). The frequency of pneumonia observed in the present study was significantly higher in Group 3 out of the three groups (*p* = 0.019). Using a partition of the chi-square method to do multiple comparisons, we found that the difference was rooted in Group 1 and Group 3 (infectious: *p* = 0.003 < 0.0125; pneumonia: *p* = 0.008 < 0.0125). On the other hand, there was no significant difference in non-infectious complications, including anastomotic fistula (*p* = 0.801) and arrhythmia(*p* = 0.353). Perioperative death was observed in only one patient in Group 3. The mortality among the three groups was comparable (*p* = 0.096) (Table 2 and Table 3).

**Table 2:** Postoperative complications and mortality.

	Group1 (*n* = 101)	Group2 (*n* = 87)	Group3 (*n* = 20)	*p* value
Postoperative complications	47	53	16	0.01
Infectious	39	44	15	0.008
Pneumonia	38	43	14	0.019
Wound infection	2	1	1	0.527
Non-infectious	11	15	4	0.353
Anastomotic fistula	6	6	2	0.801
Arrhythmia	5	9	2	0.353
Mortality	0	0	1	0.096

**Table 3:** Multiple comparison among groups (*p* value).

		Pneumonia (*p* value)	Infectious (*p* value)	Postoperative complications (*p* value)
Group1	Group2	0.103	0.1	0.049
	Group3	0.008	0.003	0.006
Group2	Group3	0.096	0.048	0.108

α’= 0.05/4 = 0.0125.

### 3.3. Postoperative Outcomes Among Group1, Group2 and Group3

There was a significant difference in the indicators of nutritional conditions including Δ’ weight, Δalbumin, Δtotal protein, Δ’ albumin, and Δ’ total protein among the three groups (Table 4). The results of further multiple comparisons showed that all the outcomes of nutritional conditions were the highest in Group 3 of the three groups and there was no significant difference between Group 1 and Group 2 except Δ’ albumin (5.79 *vs.* 7.62; *p* = 0.01 ) (Table 5). Δlymphocyte and Δ’ lymphocyte that showed the immune conditions of three groups were comparable (*p* = 0.245; *p* = 0.744). There was a significant difference among the three groups in thoracic drainage volume (*p* = 0.004). The results of further multiple comparisons indicated that the thoracic drainage volume in Group 1 was the lowest of the three groups (Group 1 *vs.* Group 2, *p* = 0.008; Group 1 *vs.* Group 3, *p* = 0.009). However, the volumes in Group 2 and Group 3 were comparable (*p* = 0.195). The first fecal passage was observed the earliest in Group 1 and latest in Group 3. LOH and hospitalization expenses were the lowest in Group 1 and the highest in Group 3.

**Table 4:** Postoperative outcomes among 3 groups.

	Group 1	Group 2	Group 3	*p* value
Δ’ weight (kg) ^b^	1 ± 2	1 ± 3	5 ± 3	0.000
Thoracic drainage volume (mL) ^b^	680 ± 562.5	920 ± 571.25	1065 ± 737.5	0.004
First fecal passage (day) ^a^	4.03 ± 0.71	4.93 ± 0.85	6.5 ± 0.95	0.000
LOH (day) ^a^	20.82 ± 3.91	23.85 ± 7.80	26.80 ± 5.11	0.000
Hospitalization expenses (yuan) ^a^	48,658.71 ± 4823.95	63,218.60 ± 16299.913	76972 ± 7132.38	0.000
Δalbumin (g L^−1^) ^a^	9.88 ± 5.01	10.30 ± 4.54	13.75 ± 5.36	0.006
Δtotal protein (g L^−1^) ^a^	13.01 ± 6.05	14.55 ± 6.62	19.46 ± 8.70	0.000
Δlymphocyte (×10^9^ L^−1^) ^a^	1.02 ± 0.44	0.99 ± 0.45	0.83 ± 0.35	0.245
Δ’ albumin (g L^−1^) ^a^	5.79 ± 4.77	7.62 ± 5.05	10.74 ± 4.32	0.000
Δ’ total protein (g L^−1^) ^a^	6.44 ± 6.04	7.05 ± 6.93	10.72 ± 6.11	0.027
Δ’ lymphocyte (×10^9^ L^−1^) ^a^	0.50 ± 0.47	0.53 ± 0.46	0.57 ± 0.34	0.744

^a^ Data and mean ± standard deviation; ^b^ Median ± range interquartile; Δ: the difference between day 3 and pre-operation; Δ’: the difference between day 7 and pre-operation; LOH: length of hospital stay.

## 4. Discussion

As the results showed in the baseline level of patients, aside from the preoperative albumin levels among the groups having a significant difference (*P* = 0.001, Group 1 = 42.3g L^−1^, Group 2 = 40.6 g L^−1^, Group 3 = 44.2 g L^−1^), there was no significant difference in the general situation of the patients, such as age, gender, pathological type, tumor location, tumor staging and the indexes reflecting the immune condition of nutrition. Surgical stress expressed by operation time and blood loss was also comparable among the three groups. However, the present study was a quasi-experimental research, and the differences before and after the operation were the key point rather than the absolute values. Therefore, the influence of the inconsistency of preoperative baseline levels on the results was small. 

**Table 5:** The results of the multiple comparison (*p* value).

		Δweight (kg)	Thoracic drainage volume (mL)	First fecal passage (day)	LOH (day)	Hospitalization expenses (yuan)
Group1	Group2	0.483	0.008	0.000	0.001	0.000
	Group3	0.000	0.009	0.000	0.000	0.000
Group2	Group3	0.000	0.195	0.000	0.047	0.000
		**Δ albumin (g L^−1^)**	**Δtotal protein (g L^−1^)**	**Δ’ albumin (g L^−1^)**	**Δ’ total protein (g L^−1^)**	**–**
Group1	Group2	0.562	0.113	0.01	0.522	–
	Group3	0.001	0.000	0.000	0.007	–
Group2	Group3	0.005	0.003	0.01	0.022	–

α’ = 0.05/4 = 0.0125.

The postoperative complications included pulmonary infection (45.67%), incision infection (1.92%), anastomotic fistula (6.73%) and arrhythmia (7.69%). Atrial fibrillation was the most common arrhythmia. There was a significant difference among the groups in terms of pulmonary infection (*p* = 0.019 < 0.05), and the multiple comparison showed that statistical differences existed between Group 1 and Group 3 (*p* = 0.008 < 0.0125, Group 1 = 80.85%, Group 3 = 87.5%). There was no significant difference among groups in the other three kinds of complications. The results were inconsistent with those of Kazuaki Kobayashi *et al*. [7]. In the research of Kazuaki Kobayashi, there was only a difference in the incidence of anastomotic dehiscence (*p* < 0.01, Group E = 33.33%; Group L = 9.84%) between the two groups and the incidence of pulmonary infection did not differ between the two groups (*p* > 0.05, Group E = 16.67%; Group L = 16.39%). The author gave the following explanation: “A possible explanation for this was that the patients in Group E received damage by preoperative chemotherapy, which was more frequently observed in Group E compared with Group L. The damage to cell recycling, vascularization, and tissue regeneration may affect the anastomotic failure, which was more frequent in Group E.” The patients in this study did not receive preoperative systemic chemotherapy and radiotherapy, so there was no difference in this aspect among the groups. Inconsistent results for the incidence of pulmonary infection of the two studies may be associated with the different grouping of the two studies. In Kazuaki Kobayashi’s study, the patients were divided into two groups: Group E contained the patients who received EN by postoperative day 3, and Group L contained the patients who received EN after postoperative day 3. However, the present study included three groups: postoperative EN started within 48 h, 48–72 h or after 72 h. The difference of the incidence of the pulmonary infection among the groups existed between Group 1 (within 48 h) and Group 3 (after 72 h). In other words, the incidence of the pulmonary infection of Group 1 was greater than that of Group 3. Consequently, the earlier enteral nutrition began, the lower the incidence of pulmonary infection would be. Moreover, decrease of incidence of pulmonary infection could not only reduce the use of antibiotics after operation, but could also shorten the duration of hospitalization and reduce its cost [24]. 

Beyond that, there were significant differences in the indicators of nutritional conditions including deviations in weight, albumin and total protein preoperatively and postoperatively among the three groups. The results of further multiple comparisons showed that statistical differences existed between Group 1 and Group 3 as well as Group 2 and Group 3, but no significant difference existed between Group 1 and Group 2 in all the outcomes of nutritional conditions except the difference in serum albumin values between day 7 and pre-operation (Δ’ albumin). In other words, the results of the two early enteral nutrition groups (Group 1 and Group 2) were better than the delayed enteral nutrition group (Group 3), but there was no significant difference between the two early enteral nutrition groups. A possible explanation for this is as follows: (1) For the outcomes used to describe the difference between day 3 and pre-operation, comparison among the three groups was equivalent to the comparison between enteral nutrition and parenteral nutrition because the patients of Group 3 received parenteral nutrition within three days after the operation. Parenteral nutrition simply led to the recovery of patients of Group 3 slower than that of patients of Group 1 and Group 2 [9]. (2) For the outcomes used to describe the difference between day 7 and pre-operation, the nutritional status of the patients in Group 3 was still worse than the patients in Group 1 and Group 2 due to the delayed start time of enteral nutrition. (3) Considering difference in the start time of enteral nutrition for Group 1 and Group 2 was only about 24 h, the indicators of nutritional conditions were not very sensitive. Consequently, there was no significant difference between Group 1 and Group 2. Moreover, the results of further multiple comparisons of first fecal passage, LOH and hospitalization expenses showed that there was significant difference in the any two groups of the three groups. More specifically, the results of Group 1 were the best of the three groups: the time for first fecal passage was the earliest, LOH was the shortest and the hospitalization expenses were the lowest. Group 2 ranked second and Group 3 ranked the last. Thus, it could be seen that the earlier enteral nutrition started, the faster gastrointestinal function recovered. Moreover, one of the factors affecting hospitalization expenses was hospitalization time. The longer the length of time, the higher the expenses, so there was an inherent relationship between the two outcomes. 

In summary, if the patients can adapt well, enteral nutrition should be started within 48 h after the operation. There are three reasons for this: (1) Although there was no significant difference between Group 1 and Group 2 in the indicators of nutritional conditions, the time for first fecal passage was earlier in Group 1, which showed that early enteral nutrition could promote intestinal function recovery. As the view that as long as intestines are functioning, we should use enteral nutrition has become popular in China, we should start enteral nutrition as early as possible. (2) Considering the economic perspective, the cost of enteral nutrition liquid was less than the parenteral nutrition solution, so enteral nutrition should be started as early as possible in order to reduce the economic burden of patients. (3) The safety coefficient of parenteral nutrition is lower than that of enteral nutrition. Parenteral nutrition easily leads to long-term catheter infection, high nutritional and metabolic disorders and respiratory and intestinal complications for patients [11]. Therefore, considering patient safety, enteral nutrition should be started as early as possible. 

Finally, under the conditions of clinical work, the present study was a quasi-experiment. Grouping was not random, so it might have led to the occurrence of bias. Especially, non randomisation of the subjects in Group 3 may affect the validity of the results. It was suggested that the experimental design of future research should be improved. Randomized controlled trials of large samples and multicenter studies are needed to provide reliable evidence for implementing early enteral nutrition safely and effectively. 

## 5. Conclusions

Early enteral nutrition within 48 h is safe and valid for postoperative esophageal cancer patients and has advantages in reducing the incidence of postoperative pulmonary infection, improving postoperative nutrition status, promoting early recovery of intestinal movement, shortening hospitalization time and reducing the cost of hospitalization. 
